# Psychometric Properties of the Smartphone Distraction Scale in Chinese College Students: Validity, Reliability and Influencing Factors

**DOI:** 10.3389/fpsyt.2022.859640

**Published:** 2022-06-16

**Authors:** Xueyang Zhao, Ting Hu, Guiyuan Qiao, Chaoyang Li, Man Wu, Fen Yang, Jing Zhou

**Affiliations:** ^1^College of Nursing, Hubei University of Chinese Medicine, Wuhan, China; ^2^Department of Tuina and Rehabilitation Medicine, Hubei Provincial Hospital of Traditional Chinese Medicine, Wuhan, China; ^3^Department of Tuina and Rehabilitation Medicine, Affiliated Hospital of Hubei University of Traditional Chinese, Wuhan, China; ^4^Department of Tuina and Rehabilitation Medicine, Hubei Institute of Traditional Chinese Medicine, Wuhan, China

**Keywords:** distraction, smartphone use, smartphone distraction scale, reliability, validity

## Abstract

**Aim:**

The objective of this study was to evaluate the Chinese version of the Smartphone Distraction Scale (C-SDS), which is an easy-to-use tool for screening the risk of smartphone distraction in Chinese college students.

**Methods:**

The C-SDS, Smartphone Addiction Scale - Short Version (SAS-SV), Fear of Missing Out scale (FoMO) and Metacognition about Smartphone Use Questionnaire (MSUQ) were used in a sample of 1,002 Chinese college students to test smartphone distraction and its influencing factors. Exploratory factor analysis (EFA) and confirmatory factor analysis (CFA) were performed to test measurement properties and factor structures of the C-SDS. Multi-variable linear regressions examined the relationships of sex, age, education level, the purpose of using a smartphone, usage of smartphone (hours per day), fear of missing out, smartphone addiction and positive and negative metacognitions about smartphone use with the C-SDS.

**Results:**

The EFA showed a 3-factor structure, which consisted of attention impulsiveness, multitasking and emotion regulation. The CFA showed that the 3-factor demonstrated an overall better model fit (*RMSEA* = 0.07, *SRMR* = 0.05, *CFI* = 0.94, *TLI* = 0.93). The C-SDS showed internal consistency (Cronbach’s α = 0.88, McDonald’s Omega ω = 0.88). Findings included that negative metacognition about smartphone use was most correlated with the C-SDS (*b* = 0.73; *p* < 0.001). Smartphone addiction, positive metacognition about smartphone use and fear of missing out also correlated with the C-SDS (*b* = 0.66, *p* < 0.001; *b* = 0.53, *p* < 0.001; *b* = 0.40, *p* < 0.001, respectively). The study shows that males compared to females (b = –1.65; p = 0.003), had a higher C-SDS score.

**Conclusion:**

The C-SDS was valid and reliable for assessing the distraction of using smartphones in the Chinese context. Being female, the purpose of using a smartphone, smartphone usage (hours per day), fear of missing out, smartphone addiction and positive and negative metacognitions about smartphone use were positively correlated to the C-SDS.

## Introduction

With the rapid development of information technology and wireless communication, people are developing an inseparable relationship with the Internet. According to a report generated by the China Internet Network Information Center in 2021, the number of mobile Internet users had reached 1.007 billion and 99.6% of them used a smartphone (1). People aged between 10 and 29 years accounted for 29.7% of all Internet users in China (1). Today, almost every college student owns a smartphone, and they use this digital medium frequently on a daily basis (2). Digital media has many convenient and positive activities in education and entertainment of college students including online searching, accessing academic resources, communicating with instructors and peers, online payments and online shopping. However, increasing concerns exist about the negative effects of long-term use and over-reliance on digital devices.

An increasing reliance on smartphones among college students may signal the evolution of smartphone use from a habit to an addiction. To date, smartphone addiction is not currently a formally accepted diagnostic construct. However, terms like “problematic smartphone use” have been used in many studies (3, 4). Problematic smartphone use (PSU) has been defined as a non-substance or behavioral addiction that results in impaired physical, mental and social functioning (5). It typically manifests as excessive usage of a phone while undertaking other activities such as studying, driving, social gatherings and even lying on the bed before bedtime (6). The PSU rate among college students in China ranges from 28.00 to 58.33% (7, 8). For college students who use smartphones, distraction has become frequent and common. Distraction is due to a lack of interest in the topic; the absence of attention; and the great intensity, novelty or attraction of something other than the object of interest (9). It derives from both internal and external sources. Internal distractions include hunger, tiredness, illness, anxiety and daydreaming. While external distractions include factors like visual triggers, social interactions, music, text messages and telephone calls (10). The smartphone has made distraction easier, due to its portability and the diversity of entertaining features. These inattention activities can have many undesirable consequences. In 2014, 47.2% of all traffic accidents in China were caused by distracted attention when using mobile phones while driving (11). Disruption from smartphone use is even more prominent within classroom environments. Available evidence suggests that smartphone use in the classroom might be an important source of distraction (12, 13). Increasing numbers of studies have shown that the use of smartphones may interrupt main tasks, further interfering with cognitive processes and ability (14, 15), cognitive functioning (e.g., thinking, memory, attention, and regulating emotions) (16, 17) and result in poor academic outcomes among college students (18).

Attention factors related to smartphone distraction are the focus of current research. Previous research has emphasized that distraction among college students was related to multitasking and executive control abilities (19). Metacognitions refer to higher order cognitive states and coping mechanisms to regulate those cognitions (20). Metacognitions can be further divided into two domains: (a) positive metacognitions about the benefits of engaging in addictive behavior as a means of cognitive and affective regulation, such as “When I get upset Smartphone use comforts me” (21); and, (b) negative metacognitions concerning the uncontrollability and dangers of thoughts and outcomes relating to the addictive behavior employed, such as “My Smartphone use persists no matter how I try to control it” (21). In recent years, the mediating role of positive and negative metacognitions between addictive behaviors and emotion regulation has also been confirmed (20–25). Metacognitive processes were chosen for construct validity due to metacognitions having been shown to play a central role in motivating individuals to participate in smartphone addictive behaviors (26). They may also serve as a potential pathway to controlling PSU (20) through positive beliefs about cognitively controlling attention (27).

According to reports, the most important interference factor when college students use smartphones is the social media platform (28). Smartphone distraction may be caused by external triggers, such as notifications. If one receives a message or call, most people will reply in time (16). The fear of missing out is a psychological state in which other people might be having rewarding experiences from which one is absent, can become an issue (29). Previous research has shown that this fear increases the desire to remain in touch with others and is the main driver of PSU (30). Problematic smartphone use reflects a prolonged pathological engagement involving use of a smartphone, which may be mediated by distraction and constant checking (31–34). With smartphone use, distraction reflects a salient cognitive and emotive coping strategy, mediating or facilitating other potentially problematic processes (e.g., checking behaviors) (31). The Smartphone Distraction Scale (SDS) not only expresses PSU behaviors, but also expresses the psychology of college students’ frequent engagement with social content (18, 35). The use of smartphone measures (metacognitions and PSU) and fear of missing out were deemed appropriate to support the validity of the C-SDS.

To the best of the researchers’ knowledge, previous studies have developed several instruments for assessing smartphone distraction. These scales are limited to a few items that assess distraction and include the mobile phone distraction scale (10) and the Social Media Disorder (SMD) scale (36). While some items can assess distraction, they are neither comprehensive nor able to assess the cognitive and emotional processes of distraction. Since there is currently no tool to measure distraction caused by using a smartphone and social media in China, Feng, S et al. measured Internet use and Facebook usage to assess distraction (37). Throuvala et al. (38) recently developed the SDS to assess smartphone distraction. The SDS includes 16 items, and factor analyses revealed a four-factor solution: attention impulsiveness, online vigilance, multitasking and emotion regulation. The author of the original version found that the SDS had good reliability and validity, and recommended further research on the factorial structure of the SDS in different populations (38). The reliability and validity of the SDS has not been tested in other populations. Given the obvious dependence of performance on attention engagement, it is important to accurately assess, identify and mitigate distractions in the context of smartphones that might capture attention and undermine performance. Therefore, the current study explored the reliability and validity of the Chinese version of the SDS (C-SDS). The purpose of this study is to evaluate the reliability, validity and influencing factors of the C-SDS in order to provide psychometric tools for evaluating distraction among Chinese college students.

## Materials and Methods

### Participants

According to the rough estimation of sample size, the number of participants needed to be five to ten times the number of items (39). Since the total number of items in the survey was 60, the sample size of this study should reach 300∼600. Taking a 20% dropout rate into account, at least an estimated 375∼750 participants are required. In this study, a relatively large sample size was investigated taking into account the diversity of the participants. Data collection began in September 2021 using offline and online methods and a total of 1,100 students were recruited from seven universities in Wuhan, Hubei Province to participate in this research. Offline data recruited 426 students through convenience sampling. Educators at these universities distributed the questionnaires and asked students to complete them in exchange for college credit. The online survey recruited 674 students and was administered by the Questionnaire Star platform. Participants were not allowed to submit the questionnaires until all questions were answered. The platform randomly allocated 50% of the participants to receive a small monetary reward. Inclusion criteria of participants were: (1) experience using a smartphone and (2) college student. The exclusion criteria were: (1) the inability to complete the online survey and (2) not reading a question carefully and answered the item in less than 3 min. The survey took approximately 7 min to complete. The final sample size was 1,002 participants after deleting invalid questionnaires with missing data.

Using SPSS 24 software, the final 1,002 participants were randomized into two sub-samples by a random number generator. The mean age in years for the total sample, sub-sample 1, and sub-sample 2 was 20.28 ± 1.54, 20.27 ± 1.60, and 20.30 ± 1.49, respectively. The first sub-sample (sample 1, *n* = 501) was evaluated using EFA, and the second sub-sample (sample 2, *n* = 501) was evaluated using CFA to assess population construct validity. The two sub-samples showed no difference in socio-demographic variables. Sample characteristics are shown in Table 1.

**TABLE 1:** Comparison between the two samples.

	Total sample (*n* = 1002)	Subsample 1 (*n* = 501)	Subsample 2 (*n* = 501)	χ^2/*t*^	*p*
Male (%)	40.02%	38.10%	41.90%	1.50	*p* = 0.221
Female (%)	59.98%	61.90%	58.10%		
Freshman	28.80%	28.30%	29.30%	4.65	*p* = 0.325
Sophomore	22.80%	25.00%	20.60%		
Junior	35.00%	32.50%	37.50%		
Senior	10.20%	10.60%	9.80%		
Postgraduate	3.20%	3.60%	2.80%		
Age (M ± SD)	20.28 ± 1.54	20.27 ± 1.60	20.30 ± 1.49	–0.27	*p* = 0.790
C-SDS (M ± SD)	49.12 ± 8.56	48.89 ± 8.57	49.34 ± 8.56	–0.84	*p* = 0.403
Attention impulsiveness (M ± SD)	23.22 ± 5.14	22.96 ± 5.06	23.49 ± 5.21	–1.64	*p* = 0.101
Multitasking (M ± SD)	12.41 ± 2.63	12.40 ± 2.71	12.41 ± 2.55	–0.07	*p* = 0.943
Emotion regulation(M ± SD)	13.49 ± 2.97	13.53 ± 3.03	13.44 ± 2.91	0.49	*p* = 0.625
MSUQ-PM	35.37 ± 8.04	35.26 ± 8.23	35.49 ± 7.86	–0.45	*p* = 0.652
MSUQ-NM	23.03 ± 5.94	22.82 ± 5.91	23.25 ± 5.96	–1.15	*p* = 0.251
SAS-SV (M ± SD)	37.35 ± 8.17	37.05 ± 8.31	37.65 ± 8.02	–1.16	*p* = 0.245
FoMO (M ± SD)	28.62 ± 8.15	28.28 ± 7.94	28.96 ± 8.35	–1.33	*p* = 0.185

*Subsample 1 = The first subsample obtained by randomly dividing the sample data into two halves; Subsample 2 = The second subsample obtained by randomly dividing the sample data into two halves. C-SDS = Chinese smartphone distraction scale; MSUQ-PM = Positive Metacognitions about Smartphone Use Questionnaire; MSUQ-NM = Negative Metacognitions about Smartphone Use Questionnaire; SAS-SV = Smartphone addiction scale-short version; FoMO = Fear of missing out scale. χ^2^/t = descriptive statistical differences of variables between sample 1 and sample 2.*

### Procedure

The SDS was authorized by the author of the original scale and independently translated into Chinese by two nursing postgraduates who had obtained a College English Test-6 Certificate. After translation, an associate professor of nursing who had a three-year visit experience in the United States reviewed the content of the scale and proposed revisions. Back translation was performed independently by researchers who spoke fluent Chinese. One was a professor of global health in the United States and the other was a doctor of nursing in the United States.

Using convenience sampling method, 38 students (44.70% female, mean age = 20.32 ± 1.21 years) from Hubei University of Chinese Medicine were selected for pre-testing and interviews to determine if the C-SDS scale was suitable for the Chinese cultural context. The students were asked if there were any unclear and difficult choices and if each item was clear and easy to understand. The Cronbach’s alpha for the total scale was 0.88 (McDonald’s Omega ω = 0.89).

### Measures

#### Socio-Demographic Characteristics and Smartphone Usage

Participants were asked their age, sex, education level and purpose of using a smartphone. According to a recent study (40), participants’ reported time of daily smartphone use was coded as follows: 1 = “less than 3 h”, 2 = “3–9 h”, 3 = “over 9 h”.

They were also asked to describe their usage of the numerous functions of smartphones, such as frequent or infrequent instant messaging, frequent or infrequent access to social media, frequent or infrequent access to music, frequent or infrequent gaming, frequent or infrequent use for learning and frequent or infrequent shopping.

#### Chinese Version of the Smartphone Distraction Scale

The 16-item SDS was developed by Throuvala et al. (38). This scale assesses the distraction of young people due to social media content, including four dimensions: attention impulsiveness, online vigilance, multitasking and emotion regulation. It uses a 5-point Likert scale, from 1 (almost never) to 5 (almost always), and the higher the score, the more distracted the user. This scale has evidenced adequate internal consistency, good reliability and validity (38). The C-SDS was a reliable measure (Cronbach’s α = 0.88, McDonald’s Omega ω = 0.88).

#### Smartphone Addiction Scale-Short Version

The 10-item SAS-SV developed by Kwon et al. (41) was used in the study, and is a self-report measure of problematic smartphone usage. Items are rated using a 6-point Likert scale ranging from 1 (strongly disagree) to 6 (strongly agree). This scale has shown effective reliability and validity in a sample of Chinese adults in Hong Kong (41). The SAS-SV showed good internal consistency (Cronbach’s α = 0.86, McDonald’s Omega ω = 0.87).

#### Metacognitions About Smartphone Use Questionnaire

The 24-item MSUQ was developed by Casale et al. (21). It uses self-report measures to assess the metacognition of addictive behaviors in using a smartphone. Items are rated on a 4-point Likert scale ranging from 1 (do not agree) to 4 (agree very much). The scale has sufficient internal consistency and validity of positive metacognition concerning emotional and cognitive regulation and social advantages of smartphone use (MSUQ-PM) and negative metacognition about the uncontrollability and cognitive harm of smartphone use (MSUQ-NM). The higher the score, the higher the degree of metacognitive dysfunction associated with the use of smartphones. The scale has good reliability and validity in a sample of college students in China (42). In the present sample, using the Chinese MSUQ, the Cronbach’s alpha for the total scale was 0.94 (McDonald’s Omega ω = 0.94), for the MSUQ-PM it was 0.94 (McDonald’s Omega ω = 0.94), and for the MSUQ-NM it was 0.90 (McDonald’s Omega ω = 0.90).

#### Fear of Missing Out Scale

The 10-item FoMO scale was developed by Przybylski et al. (29). The scale reflects current anxiety of missing out on social events and getting along with friends. Items are rated on a 4-point Likert scale ranging from 1 (not at all true of me) to 4 (extremely true of me). The scale has evidenced adequate internal consistency and good reliability and validity in multiple studies (29, 43, 44). In the present sample, the Cronbach’s alpha for the FoMO was 0.90 (McDonald’s Omega ω = 0.90).

### Data Analyses

Descriptive statistics were used to delineate the participants’ characteristics. The associations between the collected normal distribution variables were analyzed using Pearson’s correlation and non-normal distribution of data using Spearman ‘s correlation. Internal consistency was shown by a Cronbach’s alpha ≥ 0.70 (45) and McDonald’s Omega ≥ 0.70 to 0.90 (46). A value of item-total correlation > 0.4 was considered acceptable (47). The Kaiser-Meyer-Olkin (KMO) measure of sampling adequacy (KMO, > 0.80) and Bartlett’s test of sphericity (*p* < 0.05) were used to assess the suitability of the participants’ data (48). *Chi-square* test, independent sample *t*-test, assessment of skewness and kurtosis levels, EFA, convergent validity and multi-variable linear regressions were used for data analysis. All data were analyzed using SPSS 24. The CFA was performed using MPLUS 8.0. The CFA with maximum likelihood estimator was applied to test the factorial structures.

### Ethical Considerations

This study was approved by the Ethics Committee of Hubei University of Chinese Medicine (2021-ICE-015). All participants received an explanation about the purpose of the study and provided written informed consent prior to their participation.

## Results

As shown in Table 1, participants had a mean age of 20.28 (range = 17–28 years, SD = 1.54) and were primarily female (59.98%), males (40.02%). A total of 289 participants (28.80%) were freshman, 228 were sophomores (22.80%), 351 (35.00%) were juniors, 102 (10.20%) were seniors and 32 (3.20%) were postgraduates.

Participants were asked to estimate the time they spend on their smartphones each day and the smartphone applications they frequently used. Daily use time of smartphones by participants was: (3.80%) less than 3 h, more than half (69.40%) three to 9 h, and (26.80%) more than 9 h. The smartphone applications most used by participants were social media (86.90%), followed by music (73.20%), learning (40.20%), shopping (37.80%), game (36.60%) and instant messaging (22.90%).

### Item Analysis of Chinese Version of the Smartphone Distraction Scale

Table 2 shows that correlation coefficients between the C-SDS and the total score ranged from 0.47 to 0.70. All results exceeded the acceptable cut-off of 0.40, indicating statistical significant (*p* < 0.01).

**TABLE 2:** Factor loadings for the C-SDS items.

	M(SD) (*n* = 501)	Skewness (Kurtosis) (*n* = 501)	F1 (*n* = 501)	F2 (*n* = 501)	F3 (*n* = 501)	Item-total correlations (*n* = 1002)	Alpha if item deleted (*n* = 1002)
**Attention impulsiveness**							
4.I get distracted by my phone even when my full attention is required on other tasks.	2.70 (0.97)	0.07 (–0.63)	0.80			0.62**	0.88
3.I get distracted by just having my phone next to me.	2.81 (0.90)	0.29 (–0.26)	0.78			0.59**	0.88
7.I get distracted with what I could post while doing other tasks.	2.88 (0.86)	–0.03 (–0.48)	0.73			0.69**	0.87
1.I get distracted by my phone notifications.	3.25 (0.82)	–0.41 (0.14)	0.64			0.60**	0.88
2.I get distracted by my phone apps.	3.22 (0.81)	–0.27 (0.08)	0.63			0.62**	0.87
8.I get distracted thinking how many likes and comments I will get while doing other tasks.	2.63 (0.97)	0.12 (–0.57)	0.63			0.61**	0.88
6.I think a lot about checking my phone when I can’t access it.	2.86 (0.93)	0.08 (–0.49)	0.57			0.70**	0.87
5.I get anxious if I don’t check messages immediately on my phone.	2.60 (0.93)	0.37 (–0.15)	0.61			0.62**	0.88
**Emotion regulation**							
14.Using my phone distracts me from negative or unpleasant thoughts.	3.39 (0.88)	–0.21 (–0.12)		0.86		0.62**	0.87
16.Using my phone distracts me when I’m under pressure.	3.40 (0.92)	–0.28 (–0.18)		0.79		0.64**	0.87
15.Using my phone distracts me from tasks that are tedious or difficult.	3.36 (0.88)	–0.38 (0.08)		0.79		0.62**	0.87
13.Using my phone distracts me from doing unpleasant things.	3.39 (0.89)	–0.38 (–0.09)		0.78		0.63**	0.87
**Multitasking**							
12.I often talk to others while checking what’s on my phone.	2.84 (0.91)	0.13 (–0.54)			0.75	0.52**	0.88
11.I often walk and use my phone at the same time.	2.98 (0.90)	0.03 (–0.41)			0.66	0.58**	0.88
10.I can easily follow conversations while using my phone.	3.27 (0.91)	–0.30 (–0.37)			0.67	0.47**	0.88
9.I use several applications on my phone while working.	3.31 (0.87)	–0.25 (–0.20)			0.55	0.51**	0.88
Total Variance Explained (%)			58.88%				
*KMO*			0.89				
χ*2(df)*			3293.93 (120)				
*p*			< 0.01				

**p < 0.01.

### Construct Validity

#### Exploratory Factor Analysis

According to measurement of the *KMO* it was found that sampling adequacy was 0.89 for the C-SDS and Bartlett’s test of sphericity was significant χ^2^ = 3293.93 (*df* = 120, *p* < 0.01). These findings indicated that the C-SDS had common factors and was suitable for factorial analysis.

When orthogonal rotation was applied and a suppressed value of < 0.50, EFA revealed a three-factor structure solution in which all factors had an eigenvalue above 1.0. After restricting the extraction of four factor structures according to the original SDS structures, the eigenvalue of the fourth factor was 0.96. The explained variances of the three-factor and four-factor structures were 58.88% and 64.85%, respectively. Parallel analysis indicated a three-factor solution. After comparison, the three-factor structure was chosen and factor loading of 16 items was between 0.55 and 0.86 (see Table 2). It was found that the first factor (attention impulsiveness) measured the distraction of the smartphone itself and the distraction caused by checking online content, and explained 23.41% of the variance. The second factor (emotion regulation) measured distraction as an individual would use to relieve tension, stress and anxiety, and explained 19.69% of the variance. The third factor (multitasking) measured the simultaneous use of smartphone devices at work or walking, and explained 15.78% of the variance.

#### Confirmatory Factor Analysis

For the CFA, sub-sample 2 was used to compare the structural validity of the three-factor model and the four-factor model derived from the EFA conducted in sub-sample 1. In model 1, the C-SDS was defined as a three-factor model. In model 2, the C-SDS was defined as a four-factor model. To evaluate the overall model fit, Akaike Information Criterion (AIC), Bayesian Information Criterion (BIC) and conventional criteria was followed (49): *CFI* and *TLI* values of > 0.90; *SRMR* and *RMSEA* value of < 0.08 indicated a good fit. Notably, as *Chi-square* is known to be highly influenced by the sample size (50), it was not considered as a fit index in the present study. The three-factor model derived from the EFA of subsample 1 did not achieve a satisfactory fit. In model 3, a modification index (MI) that correlated item uniqueness was used for the instruments. Specifically, the uniqueness of item 1 was correlated to that of items 2, the uniqueness of item 9 was correlated to that of items 12, the uniqueness of item 14 and 16 were correlated to improve the fit indices. Finally, the modified C-SDS model 3 showed satisfactory fit indices [χ*^2^* = 309.54, *df* = 98, *p* < 0.01; *TLI* = 0.93; *CFI* = 0.94; *SRMR* = 0.05; *RMSEA* = 0.07; 90% *CI* (0.06, 0.07)] (see Table 3).

**TABLE 3:** The CFA of the C-SDS (*n* = 501).

		χ ^2^ *(df)*	*RMSEA [CI]*	*TLI*	*CFI*	*AIC*	*BIC*	*SRMR*
Model 1	3-factor model	440.84 (101)	0.08 [0.07–0.09]	0.88	0.90	17641.41	17856.46	0.06
Model 2	4-factor model	357.33 (98)	0.07 [0.07–0.08]	0.91	0.92	17563.90	17791.60	0.05
Model 3	3-factor model	309.54 (98)	0.07 [0.06–0.07]	0.93	0.94	17516.11	17743.80	0.05

*TLI = Tucher-Lewis index; CFI = Comparative fit index; RMSEA = Root mean square error of approximation. CI = Confidence Interval; SRMR = Standardized root mean square residual.*

#### Convergent Validity Analysis

Spearman’s correlation was used to analyze the correlation between the C-SDS and sex, education level, usage of smartphone (hours per day), instant messaging, social media use, music, gaming, shopping and learning applications. Pearson’s correlations between the C-SDS and age, attention impulsiveness, multitasking, emotion regulation, FoMO, SAS-SV, MSUQ-PM, and MSUQ-NM are shown in Table 4. The C-SDS scores were positively correlated with the SAS-SV scores, the MSUQ-PM, MSUQ-NM scores and the FoMO scores. Correlation coefficients ranged from 0.38 to 0.63.

**TABLE 4:** The correlations between C-SDS and other variables.

	Sex	Instant messaging	Social media	Music	Gaming	Shopping	Learning	Attention impulsiveness	Multitasking	Emotion regulation	C-SDS	FoMO	SAS-SV	MSUQ-PM	MSUQ-NM	Age	Education level	Time on smartphone
Sex	1.00																	
Instant messaging	–0.06*	1.00																
Social media	0.15**	0.08*	1.00															
Music	0.09**	0.10**	0.13**	1.00														
Gaming	–0.17**	0.13**	0.02	0.06*	1.00													
Shopping	0.26**	0.28**	0.19**	0.23**	0.15**	1.00												
Learning	0.09**	0.27**	0.08**	0.12**	0.07*	0.28**	1.00											
Attention Impulsiveness	0.08*	0.04	0.10**	0.05	0.03	0.06	–0.04	1.00										
Multitasking	0.06	0.05	0.10**	0.03	0.05	0.11**	–0.02	0.42**	1.00									
Emotion regulation	0.08*	–0.05	0.03	0.05	0.06	0.004	–0.08*	0.43**	0.45**	1.00								
C-SDS	0.09**	0.04	0.11**	0.05	0.04	0.068*	–0.07*	0.88**	0.71**	0.74**	1.00							
FoMO	–0.01	0.06	0.07*	0.02	0.08*	0.05	–0.01	0.36**	0.26**	0.25**	0.38**	1.00						
SAS-SV	0.10**	0.01	0.11**	0.08*	0.07*	0.06	–0.07*	0.61**	0.35**	0.43**	0.63**	0.32**	1.00					
MSUQ-PM	–0.03	0.00	0.04	0.06*	0.06	0.02	–0.09**	0.38**	0.34**	0.49**	0.50**	0.35**	0.43**	1.00				
MSUQ-NM	–0.07*	0.08**	0.02	0.00	0.10**	0.03	–0.03	0.52**	0.31**	0.27**	0.50**	0.44**	0.48**	0.58**	1.00			
Age	0.01	0.05	0.03	–0.08*	–0.10**	0.02	0.05	0.00	–0.04	–0.03	–0.02	–0.12**	–0.04	–0.06*	–0.07*	1.00		
Education level	0.11**	0.04	0.06	–0.04	–0.12**	0.05	0.07*	0.02	–0.03	0.01	0.00	–0.10**	–0.043	–0.06	–0.08**	0.79**	1.00	
Time on smartphone	0.05	0.00	0.02	0.10**	0.15**	0.13**	0.01	0.13**	0.16**	0.17**	0.20**	0.13**	0.19**	0.18**	0.13**	–0.11**	–0.11**	1.00

* p < 0.05, ** p < 0.01.

#### Reliability Analysis

According to some scholars, McDonald’s Omega (ω) provides more accurate reliability results for applied research (51, 52). Cronbach’s alpha (α) and McDonald’s Omega (ω) were used to assess the internal consistency of each scale. The Cronbach’s alphas of the scale were 0.88 (C-SDS), 0.87 (attention impulsiveness), 0.71 (multitasking) and 0.87 (emotion regulation). McDonald’s Omega was highest for emotion regulation (ω = 0.87), followed by attention impulsiveness (ω = 0.87), and multitasking (ω = 0.71).

#### Correlation Between Related Variables and Chinese Version of the Smartphone Distraction Scale Score

Table 5 shows that males have lower C-SDS scores than females (*b* = –1.65; 95% *CI* = –2.73, –0.58; *p* = 0.003). Compared with those who used social media infrequently, respondents who used social media frequently had higher C-SDS scores (*b* = 3.13; 95% *CI* = 1.57, 4.69; *p* < 0.001); participants who shopped frequently had higher C-SDS scores than those who shopped infrequently (*b* = 1.42; 95% *CI* = 0.33, 2.51; *p* = 0.011). This study also showed that using a smartphone for “≥ 3h/d and < 9h/d” (*b* = 3.14; 95% *CI* = 0.39, 5.89; *p* = 0.025) and “≥ 9h/d” (*b* = 6.45; 95% *CI* = 3.59, 9.31; *p* < 0.001) had a higher C-SDS score than using a smartphone for “< 3h/d”. In addition, it was found that fear of missing out (*b* = 0.40; 95% *CI* = 0.34, 0.46; *p* < 0.001), SAS-SV (*b* = 0.66; 95% *CI* = 0.61, 0.71; *p* < 0.001), positive metacognition about smartphone use (*b* = 0.53; 95% *CI* = 0.47, 0.59; *p* < 0.001), and negative metacognition about smartphone use (*b* = 0.73; 95% *CI* = 0.65, 0.80; *p* < 0.001) were related to the high risk of the C-SDS. However, it was found that age, education level, instant messaging, music, games and learning applications had no significant effect on C-SDS.

**TABLE 5:** Associations of variables with C-SDS score (*n* = 1002).

	Mean (SD) C-SDS score	b [95% CI]	*p*
**Sex**			
Female	49.78 (8.23)	Ref.	
Male	48.12 (8.96)	–1.65 [–2.73, –0.58] **	*p* = 0.003
Age		–0.12 [–0.47,0.22]	*p* = 0.486
Freshman	49.23 (8.56)	Ref.	
Sophomore	48.96 (8.27)	–0.26 [–1.75,1.23]	*p* = 0.729
Junior	49.09 (8.92)	–0.14 (–1.75,1.23)	*p* = 0.834
Senior	48.81 (8.35)	–0.42 (–2.35,1.52)	*p* = 0.675
Postgraduate	50.50 (7.76)	1.27 (–1.86,4.41)	*p* = 0.426
**The purpose of using a smartphone**			
Non- instant messaging	48.89 (8.46)	Ref.	
Instant messaging	49.65 (8.80)	0.76 [–0.40,1.92]	*p* = 0.198
Non-social media	46.40 (9.17)	Ref.	
Social media	49.53 (8.40)	3.13 [1.57,4.69] ***	*p* < 0.001
Non-music	48.28 (8.59)	Ref.	
Music	49.42 (8.54)	1.14 [–0.06, 2.34]	*p* = 0.062
Non-game	48.79 (8.44)	Ref.	
Game	49.68 (8.77)	0.88 [–0.22, 1.98]	*p* = 0.116
Non-shopping	48.58 (8.43)	Ref.	
Shopping	50.00 (8.73)	1.42 [0.33,2.51] *	*p* = 0.011
Non-learning	49.54 (8.54)	Ref.	
Learning	48.48 (8.57)	–1.06 [–2.14, 0.02]	*p* = 0.054
**Time on smartphone (hours per day)**			
< 3h	45.21 (7.37)	Ref.	
≥ 3 and < 9h	48.35 (8.38)	3.14 [0.39,5.89] *	*p* = 0.025
≥9h	51.66 (8.64)	6.45 [3.59,9.31] ***	*p* < 0.001
FoMO		0.40 [0.34,0.46] ***	*p* < 0.001
SAS-SV		0.66 [0.61,0.71] ***	*p* < 0.001
MSUQ-PM		0.53 [0.47,0.59] ***	*p* < 0.001
MSUQ-NM		0.73 [0.65,0.80] ***	*p* < 0.001

SD = Standard deviation; CI = Confidence interval; *p < 0.05. **p < 0.01. ***p < 0.001.

## Discussion

This is the first study to examine the psychometric properties of the C-SDS in a sample of Chinese college students. The C-SDS showed good internal consistency (Cronbach’s α = 0.88). A three-factor C-SDS model and a four-factor C-SDS model were also compared. However, according to model fitting indicators, the three -factor model with attention impulsiveness, multitasking and emotion regulation was considered as a better fit for evaluating smartphone distraction.

The model of this study was different from the parent version (38). The EFA results showed that attentional impulsiveness and online vigilance were on the same dimension. There are two possible reasons. First, there may be differences among the participants themselves. Second, this may be due to the distraction of the smartphone device itself and checking social media content in common. A previous study showed that college students’ use of smartphones was mainly due to checking social media content (28). Ultimately, the authors chose attentional impulsiveness as the first factor.

As a cognitive mechanism in a digital environment, distraction has been only partially evaluated in previous scales, such as the attention and executive function rating inventory scale (53) and problem Internet use scale (54). Since many existing psychometric scales are limited to a few items, they are neither comprehensive nor can they represent the complexity of a smartphone use experience or the frequent lack of attention and related processes experienced by smartphone users. For example, the recently introduced SMD scale is able to assess social media addiction. Since it was originally developed for teenagers, in the Chinese context, some items may not apply to most college students whose parents rarely supervise their smartphone use (36). The C-SDS not only assesses cognitive and emotional processes of distraction and PSU, but also applies to college students. Future research should focus on different age groups. The findings of this study supported the C-SDS as a useful tool to measure smartphone distraction in Chinese college students, which can further be used to measure the psychological experience of PSU. The C-SDS will facilitate its assessment in academic institutions and work-related environments, generating further multidisciplinary scientific knowledge about this disruptive construct and its relation to mental health in smartphone use.

The results of this study showed that fear of missing out, smartphone addiction and metacognitions about smartphone use are positively correlated with smartphone distraction. These results further support the psychopathology of smartphone distraction. In fact, earlier studies linking distraction and metacognition were based on auditory distraction (55). This finding supports recent studies that positive and negative metacognitions about smartphone use could predict PSU levels (20, 21, 42). The current study results also add to the evidence about the relationship between metacognition and distraction (38). It is worth noting that the MSUQ-NM is more strongly related to smartphone distraction than the MSUQ-PM. Previous studies used the attention control scale to assess the resistance to distraction and ability to prioritize attention. It was found that the MSUQ-NM would be negatively correlated with dimensions of attentional control (attention focusing and attention shifting) (56). This may be due to the reduced self-regulation ability of the MSUQ-NM, which further promotes distraction (27). In addition, a previous study showed that positive metacognition appears to mediate the relationship between fear of missing out and problematic social media use (25). Similarly, this result supports previous research that fear of missing out is related to PSU and social media use (57, 58). This may be due to the fear of missing out causing people to frequently keep in touch with others through social networks (57). Consistent with previous research results, smartphone addiction caused by frequent use of smartphones can distract us (59). This may be a common result of smartphone use related to cognitive interference and interruptions (60–62).

Consistent with previous studies, it was found that females were more susceptible to smartphone distraction than males (38, 63, 64). A possible reason is that women are more attached to their smartphones than males in order to establish contact and maintain social connections (63). In this study, no significant effects were found in terms of age and education level. This is inconsistent with the results of previous studies, which have shown that age was a negative predictor of PSU (41, 65). These results need to be interpreted cautiously since the current sample was composed of college students.

This study showed that increased time spent on smartphones was positively related to smartphone distraction. It is inconsistent with the previous result that the relationship between distraction and smartphone use was not significant (32). This may be due to the fact that previous studies did not measure the scale of distraction and used mindfulness measures to measure distraction through reverse scores.

The current study also found that participants who frequently used social media and shopping were more distracted than those who did not use it often. Compared with shopping, social media has a greater relationship with smartphone distractions. This may be due to the fact that social media content has largely contributed to the attention drift caused by the frequent and prolonged use of smartphones among students (66). In the future, more studies including the elderly are needed to clarify these points.

### Limitations

This study had some limitations. First, it is not certain whether the distraction scores of smartphone users are different in various age groups due to using a convenience self-selected sample of college students. Therefore, future studies should explore distraction in varied age groups, such as drivers, workers, retired older adults and clinical samples. Second, the results obtained from self-reported questionnaires may have biases of social desirability and recall. Third, the sex invariance and retest reliability of the C-SDS should be examined in future studies. Sex invariance would provide important support for the validity of the C-SDS because it would indicate that the measurement model is comparable for men and women. Lastly, more longitudinal studies or experimental studies are needed to further explore the causal relationship between smartphone distraction and metacognition for PSU.

## Conclusion

In conclusion, the C-SDS was found to be valid and reliable among Chinese college students. This study not only identified that sex, the purpose of using a smartphone, smartphone usage (hours per day), fear of missing out, PSU and positive and negative metacognitions about smartphone use were related to smartphone distraction, but also added arguments for applying distraction theory to understanding smartphone addiction. Future investigations are needed to assist in developing potential prevention programs for college students’ smartphone distraction.

## Data Availability Statement

The raw data supporting the conclusions of this article will be made available by the authors, without undue reservation.

## Ethics Statement

This study was approved by the Ethics Committee of Hubei University of Chinese Medicine (2021-ICE-015). Written informed consent to participate in this study was provided by the participants’ legal guardian/next of kin.

## Author Contributions

FY and XZ designed the study and wrote the protocol. TH, CL, and MW conducted literature searches and provided summaries of previous research studies. XZ, TH, and JZ conducted the statistical analysis. XZ wrote the manuscript. JZ and GQ provided language help. All authors have contributed to the manuscript and approved the submitted version.

## Conflict of Interest

The authors declare that the research was conducted in the absence of any commercial or financial relationships that could be construed as a potential conflict of interest.

## Publisher’s Note

All claims expressed in this article are solely those of the authors and do not necessarily represent those of their affiliated organizations, or those of the publisher, the editors and the reviewers. Any product that may be evaluated in this article, or claim that may be made by its manufacturer, is not guaranteed or endorsed by the publisher.
