# Supplementary material for: Psychometric Properties of the Smartphone Distraction Scale in Chinese College Students: Validity, Reliability and Influencing Factors
Source: Front Psychiatry. 2022 Jun 16;13:859640. doi: 10.3389/fpsyt.2022.859640 (PMC9243416; doi:10.3389/fpsyt.2022.859640)
Supplement: Supplementary file 1 [file Table_1.DOC]

Appendix A. Chinese Smartphone Distraction Scale Questionnaire

这份问卷是调查你使用智能手机的日常体验，请圈选出最能反映你的日常经验的数字来反映你的感受。请回答所有项目。答案没有对错之分。

| 题项 | 几乎从不 | 不经常 | 有时 | 经常 | 几乎总是 | 维度 |
| --- | --- | --- | --- | --- | --- | --- |
| 1.我被手机通知分散过注意力。 | 1 | 2 | 3 | 4 | 5 | F1 |
| 2.我被手机APP分散过注意力。 | 1 | 2 | 3 | 4 | 5 | F1 |
| 3.只要把手机放在身边，我就会分心。 | 1 | 2 | 3 | 4 | 5 | F1 |
| 4.当我需要全神贯注做其他事情时，我也会因为手机分心。 | 1 | 2 | 3 | 4 | 5 | F1 |
| 5.如果没有及时查看手机信息，我就会变得焦虑。 | 1 | 2 | 3 | 4 | 5 | F1 |
| 6.当我不能使用手机时，我会经常想查看手机。 | 1 | 2 | 3 | 4 | 5 | F1 |
| 7.当我在做其他事情的时候，我也会因手机上发的内容而分心。 | 1 | 2 | 3 | 4 | 5 | F1 |
| 8.当我在做其他事情的时候，我也会因我能得到多少点赞和评论而分心。 | 1 | 2 | 3 | 4 | 5 | F1 |
| 9.我在学习（工作时）会使用手机上的多个APP。 | 1 | 2 | 3 | 4 | 5 | F2 |
| 10.我可以很轻松地在使用手机的同时和别人交谈。 | 1 | 2 | 3 | 4 | 5 | F2 |
| 11.我经常一边走路一边玩手机。 | 1 | 2 | 3 | 4 | 5 | F2 |
| 12.我经常一边玩手机一边和别人聊天。 | 1 | 2 | 3 | 4 | 5 | F2 |
| 13.当我做不愉快的事情时，使用手机会分散我的注意力。 | 1 | 2 | 3 | 4 | 5 | F3 |
| 14.当我感到消极或不愉快时，使用手机会分散我的注意力。 | 1 | 2 | 3 | 4 | 5 | F3 |
| 15.当我做一些乏味或困难的事情时，使用手机会分散我的注意力。 | 1 | 2 | 3 | 4 | 5 | F3 |
| 16.当我有压力时，使用手机会分散我的注意力。 | 1 | 2 | 3 | 4 | 5 | F3 |

注：F1=注意冲动；F2=多任务处理；F3=情绪调节。
